# Microsomal Prostaglandin E Synthase-1 and -2: Emerging Targets in Non-Alcoholic Fatty Liver Disease

**DOI:** 10.3390/ijms24033049

**Published:** 2023-02-03

**Authors:** Dimitrios Kotsos, Konstantinos Tziomalos

**Affiliations:** First Propedeutic Department of Internal Medicine, Medical School, Aristotle University of Thessaloniki, AHEPA Hospital, 54636 Thessaloniki, Greece

**Keywords:** nonalcoholic fatty liver disease, nonalcoholic steatohepatitis, fibrosis, inflammation, microsomal prostaglandin E synthase 1, microsomal prostaglandin E synthase 2

## Abstract

Nonalcoholic fatty liver disease (NAFLD) affects a substantial proportion of the general population and is even more prevalent in obese and diabetic patients. NAFLD, and particularly the more advanced manifestation of the disease, nonalcoholic steatohepatitis (NASH), increases the risk for both liver-related and cardiovascular morbidity. The pathogenesis of NAFLD is complex and multifactorial, with many molecular pathways implicated. Emerging data suggest that microsomal prostaglandin E synthase-1 and -2 might participate in the development and progression of NAFLD. It also appears that targeting these enzymes might represent a novel therapeutic approach for NAFLD. In the present review, we discuss the association between microsomal prostaglandin E synthase-1 and -2 and NAFLD.

## 1. Introduction

Nonalcoholic fatty liver disease (NAFLD), a range of pathological entities that are characterized by hepatic fat aggregation, is the leading cause of chronic liver disease worldwide [1]. Globally, approximately one quarter of the total population is currently living with NAFLD, a number that is expected to rise rapidly up to 56% by 2030 in many Central European countries and the United States [1]. NAFLD, and particularly nonalcoholic steatohepatitis (NASH), is associated with increased risk for cardiovascular, liver-related, and all-cause mortality [2]. Epidemiological data suggest that in 18–33% of subjects with NAFLD, type 2 diabetes mellitus (T2DM) coexists [2]. NAFLD and NASH are emerging as the leading etiologies of hepatocellular carcinoma (HCC), the second most important cause of years of life lost due to cancer, and have been recognized as independent risk factors for chronic kidney disease (CKD) [2]. NAFLD is also associated with substantial cost for both diagnosis and management [3].

The pathogenesis of NAFLD is multifactorial, and insulin resistance, inflammation, and oxidative stress play important roles [4]. Accordingly, several therapeutic options have been evaluated in these patients, which target different pathways [5]. However, both the safety and efficacy of these treatments are suboptimal, highlighting the need for novel therapeutic strategies.

In this context, emerging data suggest that microsomal prostaglandin E synthase (mPGES)-1 and -2 might be attractive therapeutic targets in patients with NAFLD. In the present review, we summarize the role of these enzymes in the pathogenesis of NAFLD and associated comorbidities.

## 2. Search Strategy

The PubMed database was searched using the search terms: mPGES-1, mPGES1, PTGES-1, PTGES1, “microsomal prostaglandin E synthase-1”, “prostaglandin E synthase-1”, “membrane-associated prostaglandin E synthase-1”, “prostaglandin E synthase” AND liver, “prostaglandin E synthase” AND hepatocyte, mPGES-2, mPGES2, PTGES-2, PTGES2, “microsomal prostaglandin E synthase-2”, “prostaglandin E synthase-2”, “membrane-associated prostaglandin E synthase-2”. The references of pertinent articles were also hand-searched for relevant papers. Only the articles that were referring to the role of the mPGES enzymes in NAFLD, as well as to the association of the enzymes with the related comorbidities (T2DM, HCC, liver inflammation and fibrosis, liver ischemia), were considered. Moreover, articles regarding the modulation of the enzymes by potent agents were also included, along with articles about the discovery, structure, and function of the two enzymes. No limit was set regarding the period of publication.

### 2.1. Microsomal Prostaglandin E Synthase-1: Identification, Structure, Function and Expression

In 1999, Jakobsson et al. identified microsomal glutathione S-transferase 1-like 1 (MGST1-L1), a member of the Membrane-Associated Proteins in Eicosanoid and Glutathione metabolism (MAPEG) superfamily [6]. All the members of the MAPEG family have similar tertiary structures and transmembrane domains [6]. Thus, the enzyme demonstrated structural similarities and a common evolutionary origin with the other enzymes belonging to the MAPEG family (especially with MSGT1) and actually constituted the first of the three prostaglandin E synthases that were discovered [6].

**Figure 1:**
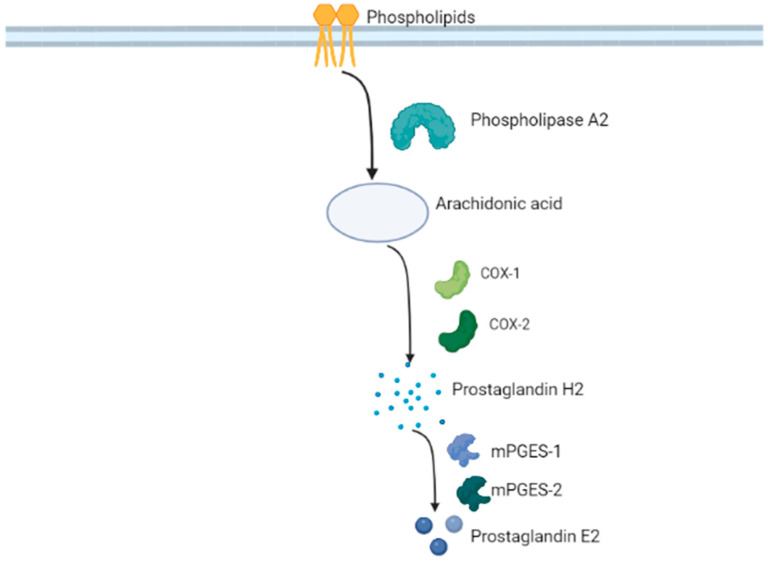
Schematic representation of the reaction catalyzed by microsomal prostaglandin E synthase-1 and -2. Their downstream position in the molecular pathway as terminal regulators of the prostaglandin E_2_ synthesis renders them ideal pharmacological targets. Figure has been created with BioRender.com (www.biorender.com, accessed on 5 January 2023).

The 17 kDa protein was initially expressed in *Escherichia coli* and possessed the activity of converting prostaglandin H_2_ (PGH_2_) to PGE_2_ (Figure 1) with strict substrate specificity [7]. In addition, it utilized PGG_2_ as a substrate to produce 15-hydroperoxy-PGE_2_ [8]. The enzyme was membrane-associated and dependent on glutathione (GSH) [8]. Thoren et al. studied its enzymatic kinetics, after expressing the protein in *E. coli* and reported a V_max_ of 170 μmol min^−1^ mg^−1^ and a k_cat_/K_m_ of 310 mM^−1^ s^−1^ [8]. The Arg^110^ residue plays an instrumental role in the enzymatic properties of the protein, as indicated by mutagenic studies that replace Arg^110^ and render it inactive [8]. In contrast, Tyr^117^ and Arg^70^ residues do not seem to be pivotal for the PGH_2_ to PGE_2_ conversion reaction [9].

The mPGES-1 gene is contained in chromosome 9q34.3, with three exons and two introns [10]. Using Northern blot analysis, it was observed that the enzyme was predominantly present in A549 and HeLa cells and to a lesser degree in several human tissues, such as the placenta, prostate, testis, mammary gland, and urinary bladder [7]. The expression of mPGES-1 is not constitutive, but activated via multiple proinflammatory mediators, such as interleukin-1β (IL-1β) [11,12,13].

### 2.2. Μ. icrosomal Prostaglandin Esynthase-1 in NAFLD Models

Whether PGE_2_ exerts a protective or a deleterious effect on hepatic lipid metabolism and NAFLD development and progression is a controversial issue, and a consensus is yet to be reached. Sterol regulatory element-binding protein 1c (SREBP-1c) is an insulin-induced transcription factor and an important activator of lipogenesis and, potentially, liver steatosis and lipotoxicity [14,15]. In hepatocyte cultures, PGE_2_ was shown to hinder the upregulation of SREBP-1c and its target molecule FAS and, hence, it was anticipated that lipid accumulation would be ameliorated. Nonetheless, it was observed that PGE_2_ actually increased fat aggregation in hepatocytes [16]. Henkel et al. showed that PGE_2_ reduced the expression of carnitine palmitoyltransferase I (CPTI), the enzyme responsible for the rate limiting step of β-oxidation [17,18], as well as the expression of apolipoprotein B (ApoB) and microsomal triglyceride transfer protein (MTP), which are both integral parts of normal very-low-density lipoprotein (VLDL) production [16,19]. The authors hypothesized that the effect of PGE_2_ on the aforementioned molecules was mediated by downregulating peroxisome proliferator-activated receptor γ coactivator 1-α (PGC1-α) [16]. PGC1-α modulates liver fatty acid oxidation, gluconeogenesis stimulation and reactive oxygen species (ROS) neutralization and is, therefore, essential for normal hepatic lipid distribution and glucose control [20,21,22]. The disruption of normal lipid metabolism by PGE_2_ in vitro is in accordance with the enhanced expression of mPGES-1 and cyclooxygenase-2 (COX-2) in obese mouse models in the same study [16].

Surprisingly, another study by Henkel et al. contradicts the aforementioned harmful effect of mPGES-1-derived PGE_2_ and suggests that it could play a protective role against liver inflammation [23]. These investigators compared mPGES-1 knockout (KO) and wild-type (WT) mice that were both fed with a high-fat diet (HFD) [23]. The diet caused lipid accumulation in the liver of both groups of mice [23]. However, the KO mice demonstrated higher levels of inflammation (increased levels of tumor necrosis factor (TNF)-α and IL-1β) [23]. Similar results were found in human subjects [23]. Hepatic tissue specimens of healthy controls and patients with hepatic steatosis were compared with patients with NASH regarding the COX-2 and mPGES-1 gene expression [23]. It was observed that both genes were overexpressed in NASH patients. Moreover, TNF-α and IL-1β mRNA were decreased in NASH patients compared to patients with steatosis [23]. It was proposed that deletion of mPGES-1 in the macrophages (both hepatic and infiltrating) deprives the cell of the autocrine inhibitory effect PGE_2_ has on TNF-α production [23]. TNF-α augments the production of IL-1β and the apoptosis of hepatocytes [24]. That being the case, targeted therapy against mPGES-1 could favor the creation of a proinflammatory hepatic microenvironment by tempering the negative feedback exerted by PGE_2_ on TNF-α and IL-1β.

Obesity is one the main driving forces of NAFLD and often coexists with diabetes mellitus and metabolic syndrome [25]. mPGES-1 interferes in multiple aspects of the metabolic syndrome and modulates, among many others, pancreatic β-cell function, and adipose tissue differentiation [26]. Even though PGE_2_ did not prompt apoptosis in HIT-T15 cells (cell lines of islets of Langerhans originating from *Mesocricetus auratus*), it did significantly disturb their normal function by suppressing cAMP levels and the PI3K/Akt axis [26]. Tran et al. reported that IL-1β leads to β-cell dysfunction through PGE_2_ [27]. Deletion of mPGES-1 in mice conferred resistance to diet-induced obesity and adipocytes inflammation, when compared to wild-type controls [28]. This finding corroborates the results of Ballesteros-Martínez, who additionally suggested that mPGES-1 deactivation creates a healthier glycemic and lipidemic profile with less insulin resistance [29]. However, the combined activity of PPARγ (peroxisome proliferator-activated receptor γ) and mPGES-1 promoted the formation of brown adipose tissue and thermogenin expression in mice, leading to more metabolically active tissue formation [30].

### 2.3. Μ. icrosomal Prostaglandin E_2_ Synthase-1 in Ischemia-Reperfusion Induced Injury

NAFLD patients display a greater vulnerability to ischemia-reperfusion injury, as indicated by the higher mortality rates of patients with steatosis after liver surgery and transplantation [31,32,33]. Impaired microcirculation, Kupffer cell dysregulation and disturbed energy metabolism due to inhibition of oxidative phosphorylation are among the responsible pathogenic mechanisms [34]. In addition, a steatotic liver has a predilection for necrosis over apoptosis in response to ischemia [35].

The Fas/Fas Ligand (Fas/FasL) system is included in the TNF superfamily and induces programmed cell death in liver cells [36,37]. When the Fas/FasL system is adequately blocked in hepatocytes, it has been shown to improve their survival and decrease ischemia-reperfusion injury and inflammation-induced tumorigenesis [38,39], as well as to lower hepatic fat accumulation by ameliorating mitochondrial respiratory function [40]. Yao et al. used transgenic mice that overexpressed mPGES-1 to evaluate its impact on the Fas/FasL axis [41]. The abundance of mPGES-1 tempered the acute liver injury caused by Fas via stimulation of the epidermal growth factor receptor/protein kinase B (EGFR/Akt) pathway [41]. Specifically, the transgenic mice exhibited less liver hemorrhage, lower serum alanine transaminase (ALT) and aspartate transaminase (AST) levels and decreased stimulation of proapoptotic agents [41].

Nishizawa et al. compared the susceptibility of mPGES-1 knockout and wild-type mice to ischemia-reperfusion injury. Knockout mice demonstrated lower ALT levels, enhanced hepatic regeneration, and infiltration by Ly6C^low^ macrophages, which improve tissue restoration, rather than by the proinflammatory Ly6C^high^ macrophages [42]. PGE_2_ produced by mPGES-1 utilizes the E prostanoid receptor 4 (EP4) to shift the transcriptional status of macrophages from tissue restorative to inflammation promoting, as indicated by the effect of PGE_2_ on bone marrow macrophages [42]. Compound III, a mPGES-1 blocker, counteracts the proinflammatory effect of the mPGES-1/PGE_2_/EP4 system and ameliorates hepatic repair [42].

### 2.4. Μ. icrosomal Prostaglandin E_2_ Synthase-1 in Liver Inflammation and Fibrosis

NAFLD encompasses a wide range of pathophysiological changes in the liver metabolism and microenvironment. Steatohepatitis, lipotoxicity, ongoing inflammation, innate immunity impairment, acute liver injury, inadequate blood supply, and fibrosis are the main pillars of disease onset and advancement [43,44,45,46]. Kupffer cells attract circulating macrophages and these in combination orchestrate NAFLD exacerbation [47]. Therefore, elucidating and harnessing the effect of macrophages could lead to novel management strategies.

Liver X receptor (LXR) is a key modulator in low-density lipoprotein (LDL) and phospholipid metabolism and in de novo lipogenesis, since it regulates the expression of genes, such as SPERP-1c, ATP-binding cassette subfamily A member 1 (ABCA1), cytochrome P450 Family 7 Subfamily A Member 1 (Cyp7A1), that are involved in these processes [48,49]. LXR and retinoid X receptors (RXR) create a heterodimer that both LXR and RXR agonists can activate. LXR blocking has been shown to alleviate NAFLD in mouse models, and the use of LXR inverse agonists, such as SR9238, could be a promising targeted therapy [50,51]. Guillem-Llobat et al. studied the effect of LXR activation on lipopolysaccharide (LPS)-stimulated macrophage cell lines [52]. The LXR ligands (25HC, TO901317, GW3965) suppressed COX-2 and mPGES-1, and consequently limited PGE_2_ production, by involving the early growth response 1 (EGR-1) and nuclear factor κB (NFκB) pathways [52]. RXR ligands (9-cis-retinoic acid) had a similar effect on PGE_2_ production [52]. Hence, LXR could be a target for NAFLD, not only as a metabolic modulator, but also as an inflammation inhibitor.

Statins have been proven to possess anti-inflammatory properties [53,54]. Physicians are often reluctant to use statins in patients with NAFLD, but many studies indicate that they could have a beneficial effect by reducing cardiovascular disease mortality and alleviating liver damage, as indicated by decreases in ALT, AST and gamma-glutamyl transferase (GGT) serum levels [55]. Simvastatin increases PGE_2_ levels in human hepatic myofibroblasts by upregulating COX-2 and mPGES-1, an effect mediated via the p38 mitogen-activated protein kinase (MAPK) pathway, geranylgeranylation blockage and GATA activation [56]. It has also been observed that overproduction of PGE_2_ can mitigate proliferation of liver myofibroblasts through cAMP [57]. Taken together, these findings suggest that statins could exert anti-fibrotic effects in addition to anti-inflammatory effects and target mPGES-1 to ameliorate fibrosis in NAFLD.

*Peroxisome proliferator-activated receptor γ* (PPARγ) has been gaining a lot of attention as a target that could harness PGE_2_ production and alleviate the severity of hepatic diseases. Liu et al. observed that hepatitis B virus x protein (HBx) increased the EGR1 mediated mPGES-1 expression in hepatocytes, a process that was greatly hampered by 15-deoxy-Δ(12,14)-prostaglandin J₂, an endogenous PPARγ agonist [58]. Ma et al. found that caffeine downregulated mPGES-1 utilizing the PPARγ-EGR1-mPGES-1 molecular pathway in HBx positive liver cells [59]. In a meta-analysis by Shen et al. examining the link between caffeine and hepatic fibrosis in NAFLD patients, it was shown that frequent intake might alleviate fibrosis in this particular group of patients [60].

Diosgenin, a dietary steroidal sapogenin [61], has been shown to have a protective role against NAFLD through modulation of multiple molecular pathways. Diosgenin upregulated AMP-activated protein kinase (AMPK) and acetyl-CoA carboxylase (ACC) and downregulated SREBP-1c and LXR in mice fed with HFD [62]. ACC catalyzes the conversion of acetyl-CoA to malonyl-CoA, and its blockage has been shown to defend the cells against NAFLD through β-oxidation stimulation and lipogenesis attenuation [63,64]. LXR impairs autophagy in hepatic cells and favors fat accumulation by upregulating autophagy-related 4B cysteine peptidase (ATG4B) and Rab-8B [65]. Tsukayama et al. administered diosgenin in mice and then induced acute liver injury using bacterial LPS and observed that the expression of mPGES-1 was repressed in sinusoidal macrophages when compared to mice that had not received diosgenin [66]. This could suggest that diosgenin could be utilized for blockage of mPGES-1 in inflammatory processes relevant to hepatic injury.

Curcumin is a polyphenol with anti-inflammatory and anti-tumorigenic properties that is widely used in traditional Chinese and Indian medicine [67,68]. In a systematic review, curcumin was shown to be a promising agent for improving liver damage in NAFLD patients [69]. Moon et al. showed that curcumin is able to impede mPGES-1 production [70]. IL-1β induces mPGES-1 via NFκB [71]. Curcumin abrogates the IL-1β-induced upregulation of mPGES-1, prevents EGR-1 from activating mPGES-1 transcription and hinders phosphorylation of NFκB inhibitor IκB (and, consequently, NFκB nuclear translocation) and of Jun N-terminal kinase (JNK) 1/2 (and, therefore, inhibiting mPGES-1 expression by cytokines) [70]. Interestingly, EGR-1 deactivation required significantly lower concentrations of curcumin, rendering it the less resistant target [70]. Likewise, the orchid *Spiranthes sinensis* limited the production of mPGES-1 in Raw264.7 macrophages that were activated by LPS, by hindering the phosphorylation of IκB [72].

Endogenous nitric oxide (NO) was the molecule responsible for the enhancement of mPGES-1 expression in rat hepatocytes, which were activated by lysophosphatidic acid (LPA) [73]. NO has an ambiguous role in the pathogenesis of NAFLD. While eNOS-produced NO tempers Kupffer cells activity and promotes β-oxidation, iNOS-produced NO exacerbates NAFLD [74,75,76].

### 2.5. Μ. icrosomal Prostaglandin E_2_ Synthase-1 in Hepatocellular Carcinoma and Cholangiocarcinoma

Hepatocellular carcinoma (HCC) is the most frequent form of primary liver cancer and the third leading cause of cancer-related mortality [77,78]. Even though hepatitis B and C viruses are the primary causes of HCC development worldwide, NAFLD and especially NASH are steadily arising as the leading etiology in western societies [79,80]. Chronic inflammation, ROS abundance, and hormonal changes are included in the pathophysiological processes that foster liver carcinogenesis [81]. mPGES-1 is upregulated in a multitude of cancer types [82,83]. PGE_2_ promotes the migration and proliferation of endothelial cells, possesses immunosuppressive properties, and prohibits immunological surveillance [84,85].

At the mRNA and protein level, HCC cells exhibit mPGES-1 overexpression in comparison to benign hepatic samples, something that renders mPGES-1 a possible contributor to tumorigenesis and tumor progression [86]. Takii et al. reported increased expression of mPGES-1 in both poorly and well-differentiated HCC cells [86]. This contradicts the findings of Nonaka et al., who found that poorly differentiated cancerous specimens had a modest mPGES-1 expression when compared to highly differentiated HCC [87]. Breinig et al. used Western blotting and discovered an overexpression of mPGES-1 and -2 in liver samples removed from patients with cirrhosis [88]. The authors hypothesized that this could be mediated by the inflammation-afflicted extracellular matrix [88]. Interestingly, in dysplastic nodules and HCC cells, the mPGES-1 levels were negatively correlated with the COX-2 levels, something that could be attributed, at least partially, to a negative feedback loop [88].

Lu et al. evaluated the effect the excess of mPGES-1 expression would have on Hep3B and Huh7 HCC cell lines [89]. It was observed that the abundance of the enzyme was an adverse characteristic that drove cellular activity toward a more malignant behavior in terms of fast multiplication, tissue penetration, and migration ability. On the other hand, cells with mPGES-1 deletion tended to be less invasive. The overexpression effect was also tested in severe combined immunodeficiency (SCID) mice xenografts, which tended to be more vulnerable to fast tumor development and higher tumor burden as opposed to mPGES-1 KO. At a molecular level, Lu et al. reported that the PGE_2_ produced by mPGES-1 prompted the activity of EGR-1 and impeded the inhibitory effect of glycogen synthase kinase-3 (GSK-3β) on β-catenin. As a consequence, the joint action of “EGR-1 and β-catenin complex” promoted malignant transformation [89].

Reduced CD8+ cell presence with a simultaneous overexpression of programmed death-ligand 1 (PD-L1) in immune cells and HCC cells was observed in HBV-induced HCC human liver specimens. In the same context, HBV-positive HCC specimens demonstrated an abundance of CD163, a T-cell immunosuppressing molecule [90]. Both 2,5-dimethylcelecoxib (DMC) (an mPGES-1 inhibitor) and atezolizumab (a monoclonal antibody that targets PD-L1 and is used in the treatment of metastatic urothelial carcinoma, triple-negative breast cancer, and non-small-cell lung cancer [91,92]) counteracted this immunosuppressive effect by attracting CD8+ cells and repressing the expression of PD-L1 and CD163, as it was shown in mice with HBx(+) HCC. The optimum effect was observed when the two agents were combined [93]. In view of the above, immunotherapeutic utilization of mPGES-1 might have a role in the future management of HCC.

Nonaka et al. investigated the relationship between mPGES-1 expression and the risk of HCC recurrence after surgical resection [87]. They observed that high expression of the enzyme in the non-cancerous liver tissue (originating from a different lobe than the HCC-afflicted one) was positively correlated with a shorter period until HCC recurrence post-operatively. Nonetheless, abundance of the enzyme in HCC tissues could not predict the recurrence-free survival rate in a similar manner [87]. This phenomenon could be explained as follows: the excess production of PGE_2_ due to mPGES-1 overexpression facilitates the generation of an inflammatory microenvironment that harbors the malignant cells and allows them to proliferate, and perhaps migrate even further, in the presence of tumorigenic mitogens and oxidative stress [87]. mPGES-1 could, hence, be exploited not only as a therapeutic target in HCC, but also as a prognostic factor.

Cholangiocarcinoma (CCA) is a heterogeneous entity that encompasses many epithelial cancers and is characterized by a particularly poor prognosis and diagnosis in the very late stages of the disease [94,95]. NAFLD, diabetes mellitus, and obesity are all considered risk factors for CCA [96,97]. mPGES-1 is upregulated in human CCA tissues, and its expression is markedly higher than in normal biliary epithelial cells [98,99]. Jongthawin found a positive correlation between strong expression of the protein in CCA cells and advanced cancer (III and IV stage, spread to the lymph nodes, lower survival rates) [99]. The authors then used an mPGES-1 inhibitor (CAY10526) on CCA cell cultures, which repressed proliferation and metastatic potential [99]. Lu at al. developed in vitro cell lines of CCA cells with deletion of mPGES-1, which demonstrated no irregular proliferation patterns, as opposed to cell lines overexpressing the enzyme, which exhibited uncontrolled multiplication [98]. In parallel, SCID mouse models that overexpressed the protein had rapid and aggressive tumor progression, whereas mPGES-1 KO mice had a much less aggressive phenotype [98]. The tumorigenic potential of mPGES-1 was attributed toEGR-1 obstruction from upregulating phosphatase and tensin homolog (PTEN) [98]. This inhibition results in the EGRF-PI3K-AKT-mTOR axis stimulation that promotes CCA development [98].

Table 1 summarizes the effects of mPGES-1 in the pathogenesis of NAFLD and related liver diseases. Table 2 summarizes the agents that modulate the activity of mPGES-1.

### 2.6. Microsomal Prostaglandin E Synthase-2: Identification, Structure, Function and Expression

Microsomal prostaglandin E synthase-2 (mPGES-2) was first detected in the heart, spleen and uterus of rat tissues and purified from bovine heart microsomes in an N-terminally truncated form, as described in two seminal papers by Watanabe [100,101]. This was followed by the characterization of the enzyme by the same group, which identified cDNAs encoding human and monkey homologs [102]. The truncated and full-length mPGES-2 forms were produced in *Escherichia coli*, and their enzymatic activities were similar to each other and to the bovine purified synthase [102,103]. mPGES-2 is originally produced as a precursor, Golgi membrane-associated protein that subsequently undergoes proteolytic cleavage of the N-terminal region to obtain its active form and is thereafter distributed to the cytosol [104]. The full-length enzyme contains 87 additional hydrophobic N-terminal amino acid residues that firmly stabilize it on the membrane [102].

With regard to its catalytic function, mPGES-2 is a 33 kDa, GSH-independent enzyme (GSH can be substituted by other SH-reducing agents) that converts cyclooxygenase (COX)-produced PGH_2_ to PGE_2_ [102] (Figure 1). Nevertheless, it has been reported that mPGES-2 also binds with heme and GSH to catalyze the degradation of PGH_2_ to 12 (S)-hydroxy-5,8,10 (Z,E,E)-heptadecatrienoic acid (HHT) and malondialdehyde (MDA), without the production of PGE_2_, which renders it the first case of a “dual-function enzyme” [105,106]. The active site of mPGES-2 has the sequence ^110^Cys-x-x-Cys^113^, a consensus sequence present in the active sites of glutaredoxin and thioredoxin. An induced mutation affecting only ^110^Cys or both ^110^Cys and Cys^113^ substantially attenuated its enzymatic activity, whereas the mutation affecting the Cys^113^ alone had a minor impact. Therefore, Watanabe et al. suggested that ^110^Cys is crucial for the isomerization of PGH_2_ to PGE_2_ [103]. mPGES-2 enzymatic activity was increased by GSH, 2-mercaptoethanol and Coenzyme A (CoA), but the greatest cofactor was proved to be dithiothreitol [103].

In the human genome, the gene for mPGES-2 is contained in chromosome 9q33-34, a locus that is closely related to prostaglandin metabolic processes, since it also contains COX-1 and lipocalin-type PGD synthase genes and is linked to obesity and body weight [10,102,107,108]. mPGES-2 is constitutively expressed in many tissues, predominantly in the kidneys, liver, heart, and brain [102,109,110].

### 2.7. Μ. icrosomal Prostaglandin E_2_ Synthase-2 in NAFLD Models

To elucidate the contribution of mPGES-2 to NAFLD, Zhong et al. compared the effect of HFD on mPGES-2 knockout and wild-type mice. The knockout mice demonstrated lower NAFLD activity score, less severe hepatic inflammation, fat accumulation and fibrosis, lower plasma ALT and AST levels, and reduced liver weight to body weight ratio [111]. Given that diabetes mellitus is a major risk factor for NAFLD development and progression to NASH, fibrosis, and cirrhosis [112,113], Zhong et al. attempted to further investigate the effect that the absence of mPGES-2 would have on db/db diabetic mice [114]. Thus, db/db diabetic mPGES-2 KO mice were compared to db/db diabetic mice with intact mPGES-2 [114]. The results corroborated those of the first experiment in terms of liver histology, with the knockout mice having lower serum and liver triglyceride (TG) levels [114]. The same results were observed in mice when fed with a methionine-choline-deficient (MCD) diet, a diet that induces liver steatosis [115,116], with ameliorated lobular inflammation and liver ballooning [114].

Nuclear receptor subfamily 1 group D member 1 (NR1D1) belongs to the nuclear receptor subfamily, is abundant in liver and adipose tissue, and participates in energy metabolism [117,118]. The active NR1D1-heme complex induces the expression of acyl-CoA thioesterase 4 (ACOT4) by negatively regulating E4bp4 and at the same time inhibits the expression of CYPA414. ACOT4 belongs to the family of ACOTs and catalyzes the hydrolysis of fatty acyl-CoA to CoA-SH and free fatty acids [119]. mPGES-2 deletion allows for higher levels of heme binding with NR1D1 and, therefore, leads to promotion of ACOT4 expression, which alleviates fat accumulation in the liver [111]. The protective role of ACOT4 against steatosis has also been reported to be induced by other molecules, such as microRNA-23b [120]. Conversely, mPGES-2 deletion downregulates the expression of CYPA414, which has been shown to ameliorate lipid accumulation and liver fibrosis [111]. Those results indicate that a lack of mPGES-2 can mitigate the deleterious effect an HFD has on the liver, and that those effects are mediated via the NR1D1 interaction with ACOT4 and CYPA414. The clarification of the contribution of mPGES-2 to the pathogenesis of NAFLD could facilitate the development of molecular inhibitors of the enzyme and enable a paradigm shift toward targeted therapy for NAFLD. Indeed, SZ0232, an inhibitor of mPGES-2, alleviated liver injury and lipid aggregation in mice [111,114].

As mentioned above, the mPGES-2 gene is in close proximity to genes related to weight gain. PGE_2_ hinders lipolysis and promotes adipocyte growth [121]. In two German cohorts, Nitz et al. reported that the recessive Arg298His allelomorph of mPGES-2 confers protection against type 2 diabetes mellitus in heterozygous individuals [122].

### 2.8. Microsomal Prostaglandin E_2_ Synthase-2 in Drug Toxicity and Inflammation

Wang et al. reported that acetaminophen (APAP)-induced liver injury 12 and 24 h after APAP administration was significantly decreased in mPGES-2 KO mice, as they exhibited less liver inflammation (lower TNF-α, IL-1β, IL-6, monocyte chemoattractant protein-1 (MCP-1) and NLR family pyrin domain containing 3 (NLRP3) liver levels) and less severe histological abnormalities [123]. A postulated mechanism for this phenomenon could be the upregulation of GSH (in the absence of mPGES-2 that results in reduction of MDA, a molecule that leads to GSH depletion) that was observed in the knockout mice. GSH is a detoxification factor that protects against N-acetyl-p-benzoquinone imine (NAPQI), a hepatotoxic molecule produced by APAP [124,125]. At the same time, a decrease in the APAP-CYS was observed, which is a measure of the hepatotoxic NAPQI molecule produced by APAP, which further confirms the GSH-related mechanism of protection [126].

Streptozotocin (STZ) is an antibiotic that can lead to destruction of the β-cells in the pancreas and is, therefore, used for type 1 diabetes mellitus induction in animal models [127]. Streptozotocin uses the glucose transporter-2 (GLUT2) receptor to invade β-cells and causes alkylation of DNA [128]. Sun and Jia et al. tested the effect of STZ administration on mPGES-2 KO and WT mice [129]. The knockout mice were less resistant to STZ-induced liver injury when compared to wild-type and tended to develop acute liver failure, with high ALT and AST levels, and hepatic steatosis and inflammation (TNF-α, MCP-1, IL-1β) [129]. In addition, multiple pro-apoptotic genes were overexpressed (caspase 3, BAK, BAX) [129]. The high sensitivity of knockout mice to STZ could be attributed to the upregulation of GLUT2 devoid of mPGES-2 [129]. The authors speculated that the insulin/SREBP-1c pathway was responsible for the overexpression of GLUT2, since the knockout mice had higher levels of insulin and SREBP-1c (which prompts GLUT2 expression) production [129]. Hence, both deletion and induction of mPGES-2 can be desirable, depending on the tissue concerned and the effect the enzyme exerts.

Anti-inflammatory therapeutic interventions can be enriched by agents that block mPGES-2 in immune cells. Isoquercitrin, a flavonoid derived from green ball apple peel, has been shown to suppress mPGES-2 (and subsequently reduce PGE_2_) in Raw 264.7 macrophages [130]. In this way, it could potentially be utilized as an anti-inflammation agent. Curcumin is a promising agent against NAFLD and hyperlipidemia (that, as mentioned above, can also affect mPGES-1), but is characterized by a limited ability to enter the systemic circulation. In contrast, mCurc-mPEG454 is a product of curcumin pegylation with a much more favorable bioavailability profile [131]. Both COX-2 and mPGES-2 expression were downregulated by mCurc-mPEG454, which led to a substantial reduction in hepatic PGE_2_ expression in rodents [132]. *P. altissima* extract restricted the production of PGE_2_ by hindering the expression of all three prostaglandin E synthase enzymes in monocytes activated by LPS [133]. A recently manufactured mPGES-2 inhibitor, 2-chloromethylquinoline, is also a promising agent against hepatic inflammation [134].

Table 3 summarizes the effects of mPGES-2 in the pathogenesis of NAFLD and related liver diseases. Table 4 summarizes the agents that modulate the activity of mPGES-2.

## 3. Conclusions

Both mPGES-1 and -2 appear to play a role in the pathogenesis of NAFLD. Accumulating evidence also suggests that experimental treatments that target these enzymes might ameliorate hepatic histology in models of NAFLD. It remains to be established whether these findings will translate into humans and whether this therapeutic approach will delay the progression of NAFLD.

The multifactorial pathophysiology of NAFLD, the structural variance of mPGES between humans and mice, and the heterogeneity of the effects of mPGES in cell cultures and animal models, are among the main obstacles to the translation of the preclinical model findings to clinical practice. Further research is needed to elucidate the exact role of mPGES-1 and -2 in liver fat metabolism and inflammation. Whether the enzymes will induce or reduce inflammation strongly depends on the tissue and the cytokines involved. Moreover, some pathological entities would be ameliorated by the induction of the enzyme, while others by its suppression. Clarification of the pathways and mechanisms implicated will provide the chance to manipulate the enzyme using targeted therapy and exploit it optimally.

Conventional non-selective non-steroidal anti-inflammatory drugs (NSAIDs) are commonly used as blockers to inhibit PGE_2_ synthesis, but their use is associated with serious side effects, such as gastrointestinal bleeding, as well as cardiovascular and renal complications. In addition, they unnecessarily affect the production of multiple other bioactive lipids [135,136]. Similarly, selective COX-2 inhibitors have been linked to cardiotoxicity [137]. In this context, mPGES enzymes, being the terminal regulators of PGE_2_ production, could constitute a superior target, providing the opportunity for a more selective blocker, with potential cardioprotective properties [137]. Obeticholic acid is a selective ligand and activator of the farnesoid X receptor, that, when combined with statins, ameliorated the lipidemic profile and lowered the NAS score of patients with NASH [138]. Elafibranor, a PPAR-α and PPAR-δ activator, led to resolution of NASH, improved insulin resistance and serum lipid levels, but also caused an increase in creatinine levels [139,140]. With so many new experimental therapies being tested in the field of NAFLD, it appears plausible that mPGES modulators will also become available in clinical practice in the future, offering a favorable safety profile. Another interesting approach for the development of mPGES inhibitors is the repositioning of drugs, with a multitude of FDA-approved drugs being tested as potential mPGES blockers. One current example is a drug reported by Zhou et al., lapatinib, an anti-cancer kinase inhibitor that was proved to be a potential mPGES-1 inhibitor and a promising agent against inflammation and pain [141].

## Figures and Tables

**Table 1:** Effects of microsomal prostaglandin E synthase-1 in the pathogenesis of nonalcoholic fatty liver disease and related liver diseases.

Effect on Liver Histology	Implicated Molecular Pathway
Aggravation of steatosis	Inhibition of SREBP-1c
	Reduced expression of CPTI
	Downregulation of PGC1-α
Inhibition of inflammation	Decreased production of TNF-α and IL-1β
Alleviation of Fas-induced liver injury	Upregulation of the EGFR/Akt pathway
Aggravates ischemia induced injury	Utilization of EP4 receptor to shift the transcriptional status of macrophages from tissue restorative to inflammation promoting
Favors malignant transformation and more aggressive HCC phenotypes	EGR-1 and β-catenin complex activation
Favors CCA proliferation	EGRF-PI3K-AKT-mTOR axis stimulation

CCA: Cholangiocarcinoma, CPTI: Carnitine palmitoyltransferase I, EGFR/Akt: Epidermal growth factor receptor/protein kinase B, EGR-1: early growth response 1, EP4: E prostanoid receptor 4, HCC: Hepatocellular carcinoma, IL-1β: Interleukin-1β, mTOR: Mammalian target of rapamycin, PGC1-α: peroxisome proliferator-activated receptor γ coactivator 1-α, PI3K: phosphatidylinositol 3-kinase, SREBP-1c: Sterol regulatory element-binding protein 1c, TNF-α: Tumor necrosis factor-α.

**Table 2:** Agents that modulate microsomal prostaglandin E synthase-1.

Agent	Molecule/Pathway Modulated	Effect on Liver Histology/Immune Responses
Compound III	Inhibition of the mPGES-1	Improved hepatic repair
25HC, TO901317, GW3965, 9-cis-retinoic acid	Suppression of LPS mediated mPGES-1 expression by LXR/RXR activation	Lower PGE_2_ production by macrophages
Statins	Upregulation of mPGES-1 in liver myofibroblasts	Potential antifibrotic effects
Caffeine	Downregulation of mPGES-1 via the PPARγ-EGR-1-mPGES-1 axis	Potential antifibrotic effects
Diosgenin	Suppression of mPGES-1	Potential attenuation of inflammation related to acute liver injury
Curcumin	Suppression of mPGES-1 via inhibition of activation by NFκB and EGR-1	Improved liver damage
*Spiranthes sinensis*	Suppression of mPGES-1 by hindering the phosphorylation of IκB	Improved liver damage
2,5-dimethylcelecoxib	Inhibition of mPGES-1	Counteracted immunosuppression in HCC cells
CAY10526	Inhibition of mPGES-1	Repressed proliferation and metastatic potential in CCA cells

CCA: Cholangiocarcinoma, EGR-1: early growth response 1, HCC: Hepatocellular carcinoma, LXR/RXR: Liver X receptor/Retinoid X receptor, mPGES-1: Microsomal prostaglandin E synthase-1, NFκB: Nuclear factor κB, PGE_2_: prostaglandin E_2_, PPARγ: peroxisome proliferator-activated receptor γ.

**Table 3:** Effects of microsomal prostaglandin E synthase-2 in the pathogenesis of nonalcoholic fatty liver disease and related liver diseases.

Effect on Liver Histology	Implicated Molecular Pathway
Protection against steatosis	Induction of the expression of ACOT4
	Downregulation of the expression of CYPA414
Protection against inflammation and fibrosis	Upregulation of glutathione
Higher resistance to streptozotocin-induced injury	Upregulation of GLUT2 in the absence of mPGES-2

ACOT4: Acyl-CoA thioesterase 4, GLUT2: Glucose transporter-2, mPGES-2: Microsomal prostaglandin E synthase-2.

**Table 4:** Agents that modulate microsomal prostaglandin E synthase-2.

Agent	Molecule/Pathway Modulated	Effect on Liver Histology/Immune Responses
SZ0232	Inhibition of mPGES-2	Alleviated liver injury and lipid aggregation
Isoquercitrin	Suppression of mPGES-2	Potential anti-inflammatory properties
mCurc-mPEG454	Suppression of mPGES-2	Decrease in PGE_2_ levels
*P. altissima*	Suppression of all three prostaglandin E synthase enzymes	Potential anti-inflammatory properties
2-chloromethylquinoline	Inhibition of mPGES-2	Potential anti-inflammatory properties

mPGES-2: Microsomal prostaglandin E synthase-2, PGE_2_: Prostaglandin E_2_.

## Data Availability

Not applicable.
